# PhyloToAST: Bioinformatics tools for species-level analysis and visualization of complex microbial datasets

**DOI:** 10.1038/srep29123

**Published:** 2016-06-30

**Authors:** Shareef M. Dabdoub, Megan L. Fellows, Akshay D. Paropkari, Matthew R. Mason, Sarandeep S. Huja, Alexandra A. Tsigarida, Purnima S. Kumar

**Affiliations:** 1 Division of Periodontology, College of Dentistry, The Ohio State University, Columbus, Ohio, USA; 2 Division of Biosciences, College of Dentistry, The Ohio State University, Columbus, Ohio, USA; 3 Division of Orthodontics, College of Dentistry, University of Kentucky, Lexington, Kentucky, USA; 4 Division of Periodontics, Eastman Institute for Oral Health, School of Medicine and Dentistry, University of Rochester, Rochester, NY, USA

## Abstract

The 16S rRNA gene is widely used for taxonomic profiling of microbial ecosystems; and recent advances in sequencing chemistry have allowed extremely large numbers of sequences to be generated from minimal amounts of biological samples. Analysis speed and resolution of data to species-level taxa are two important factors in large-scale explorations of complex microbiomes using 16S sequencing. We present here new software, Phylogenetic Tools for Analysis of Species-level Taxa (*PhyloToAST*), that completely integrates with the QIIME pipeline to improve analysis speed, reduce primer bias (requiring two sequencing primers), enhance species-level analysis, and add new visualization tools. The code is free and open source, and can be accessed at http://phylotoast.org.

Understanding human-associated microbial ecology is essential for insight into health, as well as identifying disease states, risk factors, and etiology. The 16S ribosomal RNA gene is the most common genetic marker for bacterial taxonomic identification due to its near universal presence, static function over time, and mix of highly conserved and hypervariable regions^1^ which allow for species-specific identification of samples without relying on culturing^2^. As a result, a wide variety of tools exist for quantifying samples of 16S data. The widely popular QIIME (Quantitative Insights Into Microbial Ecology)^3^ software was developed to gather such tools into a single coherent pipeline; organizing operational taxonomic unit (OTU) data into per-sample abundance, in addition to phylogenetic tree creation and computation of useful statistics. However, we have found areas of improvement in processing time and species-level analysis, which is of particular importance in examining highly complex human microbiomes. To remedy these issues, we have developed new software, Phylogenetic Tools for Analysis of Species-level Taxa (PhyloToAST), that wholly integrates with the QIIME pipeline.

The genesis of this project grew from our day-to-day work process and the need address gaps between the tools provided by QIIME and our analysis needs. The first important contribution provided by PhyloToAST is the ability to distribute BLAST-based OTU picking across computing clusters. Each sequence is independent from every other sequence in this process, making it possible to split the input data into any number of smaller files to be processed separately. After OTU picking, the resulting OTU-assignment files are merged back together into a single comprehensive file for the whole dataset. The second highly useful tool is a process for eliminating redundant taxonomic information from the assigned OTUs. Public databases, such as GreenGenes, often contain many entries for the same species. The common enteric species *Escherichia coli*, for example, has 44 separate entries in the Greengenes database. Without additional classification such as subspecies or serovars, these entries clutter visual representations of the data, and do not provide meaningful information in understanding sample diversity or making statistical comparisons. The taxonomy condensing workflow provided by PhyloToAST groups these redundant OTUs into single bins representing the desired phylogenetic level.

At a broader level, PhyloToAST provides several improved/new visualization methods, tools for filtering and subsetting results files, simple name lookup for OTU IDs, and finally, exposes the API used to build all of these tools for interested developers.

## Results

**Figure 1:**
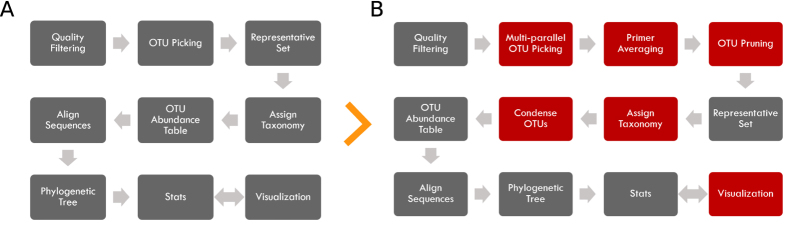
Changes between the original QIIME pipeline analysis process and the new PhyloToAST software package. (**A**) QIIME pipeline. (**B**) The new pipeline including steps in red modified or added by PhyloToAST.

Since beginning development on PhyloToAST, the project has been used to analyze a number of investigations, and continues to be used for ongoing and upcoming work. Here we present three different investigations that made use of our integrated QIIME/PhyloToAST pipeline to perform species-level analyses of oral microbiome data. Figure 1 provides an overview of the process changes introduced by PhyloToAST.

### Data Processing and Analysis

#### Case Study 1: Improved visualizations for beta diversity

Smoking is well established as a major risk factor for the development of periodontal diseases, and emerging evidence indicates that this is due in part to smoking-induced alterations in the subgingival ecosystem and subsequent changes to microbial colonization dynamics^4^,^5^. Furthermore, smoking is known to alter host-bacterial interactions as well as inter-bacterial interactions such as aggregation and microbial gene expression^6^,^7^. Together these factors increase risk for disease in smokers; and this study examined whether smoking influences the health-associated subgingival microbiome. To this end, subgingival samples were obtained from 100 never smokers and 100 current smokers, whole-cell DNA extracted^8^ and subjected to two-region 16S pyrosequencing as described in the Methods. After quality filtering and chimera depletion, more than 1.5 million high-quality sequences were classified to species-level operational taxonomic units (s-OTUs)^9^.

**Figure 2:**
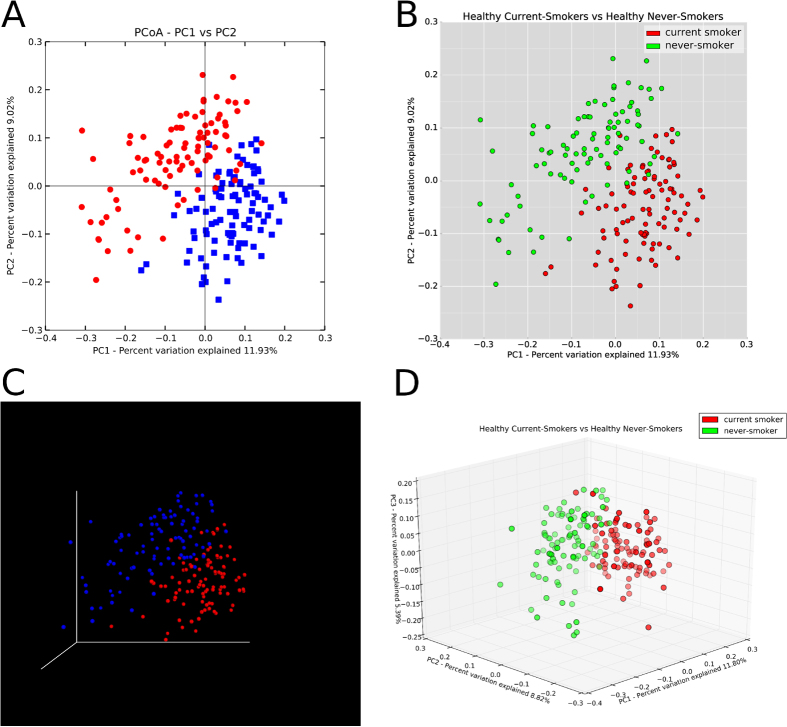
Beta diversity represented by Principle Coordinate Analysis plots of never and current smokers produced using default options by QIIME ((**A**,**C**) (Emperor plot)) and PhyloToAST (**B**,**D**). Each plot point represents a single study subject, with distances between subjects determined by the UniFrac metric. The PCoA plotting tool provided by PhyloToAST allows users to specify any combination of metadata columns and values from a mapping file to define groups that will be plotted with user-supplied colors. 2D (**B**) and 3D (**D**) plots are available through the same tool, providing greater simplicity and visual consistency over QIIME, which requires separate programs to produce each plot. Furthermore, PhyloToAST allows users to specify angle and azimuth for 3D plotting, enabling greater control and reproducibility.

Shannon Diversity (species richness and abundance, see Methods) and Equitability (contributions of richness and evenness to diversity) indices revealed significantly higher diversity in smokers, especially with regard to anaerobic species, suggesting that smokers are deficient in niche saturation capability. At both the genus (*Enterococcus, Fusobacterium, Filifactor, Pseudomonas*, and *Veillonella* in favor of smokers) and species level, significant differences were found. Differences in microbial composition between never and current smoker groups were quantified as an all-pairs distance using principle coordinate analysis (PCoA) of the unweighted UniFrac distances as a metric. The resulting graph shows striking visual difference between the communities (Fig. 2). The PhyloToAST-produced PCoA (Fig. 2B,D) were created using the same script (PCoA.py), specifying a 2D or 3D plot respectively. The PCoA plots created by QIIME (Fig. 2A,C) require two separate programs (make_2d_plots.py and make_emperor.py) and colors and legends can only be specified afterward (3D Emperor plot) or manually added/modified with an image editing program (2D plot). In addition, PhyloToAST enables easy reproducibility by displaying the azimuth and angle during the interactive 3D plotting mode, and allowing users to input those values in later sessions.

**Figure 3:**
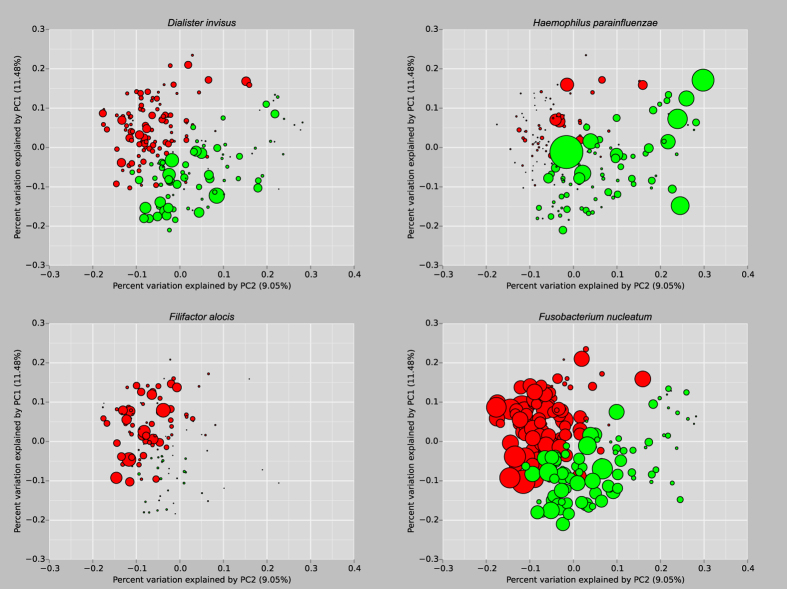
Principle Coordinate Analysis plots of never (green) and current (red) smokers (produced by the PhyloToAST script PCoA_bubble.py). Each plot point represents a single study subject, scaled by relative abundance of the named species in the plot title^9^. Plots on top demonstrate species more highly abundant in non-smokers; below in smokers.

Using PhyloToAST, species with significant differences were represented with the UniFrac/PCoA metric using the bubble-plot tool (a small selection is presented in Fig. 3). Of such species, note that the smokers have a monopoly on a putative periodontal pathogen, the anaerobic *Filifactor alocis* (whose growth is stimulated under oxidative stress), and significantly higher abundance of *Fusobacterium nucleatum*; a promiscuous co-aggregator and an important bridge species. These results indicate that smoking creates a diverse, pathogen-rich/commensal poor, highly anaerobic community that is more similar to the microbiomes found in disease than health^9^.

#### Case Study 2: Visualizing alpha diversity through kernel-smoothed histograms and beta diversity through Linear Discriminant Analysis

Animal models of periodontitis have routinely been used to understand pathogenesis of disease. Traditional methodologies for inducing disease have included infecting these animals with human periodontal pathogens and observing the clinical and immunological changes that occur in response to these infections. However, it is unlikely that infection with human pathogens elicit the same immune responses in an animal as in a human. The rice rat (*Oryzomy palustris*) is known to exhibit naturally occurring periodontitis, with bone loss and pathologic migration of teeth^10^. The aim of this study was to characterize the oral microbiomes associated with periodontal health and disease in *Oryzomy palustris* and to compare these with the human subgingival microbiome in health and disease.

**Figure 4:**
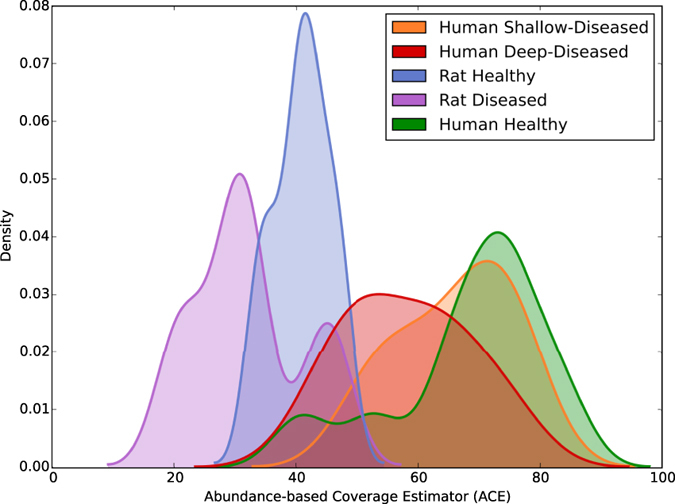
PhyloToAST-generated (diversity.py) alpha diversity (ACE) histograms of each of the five groups, smoothed by kernel density estimation. The diversity of the Rat Diseased samples are significantly different from both the Human Deep-Diseased samples (*p* < 0.008) and the Human Shallow-Diseased samples (*p* < 0.008), but not the Rat Health samples (*p* < 0.222). Furthermore, the Rat Health samples are significantly different from the Human Health samples (*p* < 0.013). Pairwise comparisons carried out by Wilcoxon Rank Sum, FDR < 0.1.

**Figure 5:**
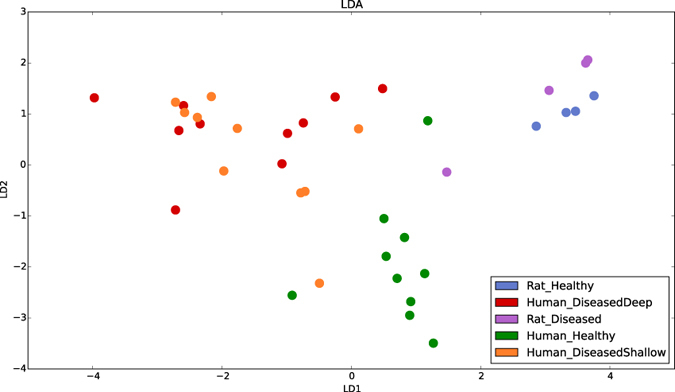
Linear Discriminant Analysis (LDA) showing clear clustering by microbial composition between the five groups. Notably, the rat samples are quite different from all of the human samples. In addition, the close clustering of the rat samples indicates that there is very little difference in composition between health and disease in rats; a feature not seen in the human samples. LDA plots were generated by PhyloToAST (LDA.py).

Examination of group-wise diversities (calculated using the Abundance-based Coverage Estimator (ACE) method^11^) reveals the first indication that, in terms of the microbiology, rats may not be an accurate model for human periodontitis. The PhyloToAST-generated curves in Fig. 4 clearly demonstrate that, as compared to the human subjects, the rat samples are shifted to the lower side of the diversity scale. By pairwise statistical comparisons (Wilcoxon Rank Sum, FDR corrected) we see that both the Rat Disease and Rat Health samples are significantly different from all of the human samples. Using PhyloToAST to visualize beta diversity through Linear Discriminant Analysis (LDA) of sample-wise compositional data, we see similar results (Fig. 5). The rat samples clearly cluster very closely and, while not overlapping, are not as distinctly separate as the healthy and diseased human samples. Through examination of the species-level abundance data (Supplementary Fig. S1) it becomes clear that the main reason for these differences is the lack of common human oral species in the rats. Genera including *Limnohabitans, Pelomonas, Paucibacter, Methyloversatilis, Methylobacterium*, and *Stenotrophomonas* were uniquely found in Rat samples, while common Human-associated genera such as *Fusobacterium, Prevotella, Porphyromonas, Tannerella, Prevotella, Treponema, Aggregatibacter, Acinetobacter, Rothia, Desulfobulbus, Peptostreptococcus*, and *Bulleidia* are not found in the Rat samples. These results are unsurprising given what is known about the influence of environment and host genetics on the oral microbiome^9^,^12^. In addition, periodontitis in the Rice Rat is highly dependent on diet^13^ and can be transmitted through coprophagy^14^. Together these data bring into question the suitability of the Rice Rat as a model organism for human periodontitis.

#### Case Study 3: Quantitative visualization of phylogentic trees

Through this comparative analysis of adjacent periodontal and peri-implant microbiomes, we introduce the PhyloToAST tool for visualizing phylogenetic trees with quantitative data. Placement of dental implants is an increasingly common procedure, with 400,000 being placed each year and a projected growth of 9.1%^15^. In the etiology of peri-implant diseases, periodontal biofilms have been presumed to be microbial reservoirs for nearby implants. However, many investigations into other oral microbial communities (including the investigation on smokers described here) indicate that the ecology of a local environment contributes greatly to the stability and composition of biofilms. Indeed, emerging evidence indicates that the peri-implant crevice is quite biologically distinct from the subgingival sulcus, and this study was the first to fully characterize the degree of similarity of adjoining periodontal and peri-implant microbiomes in varying states of health and disease^16^.

**Figure 6:**
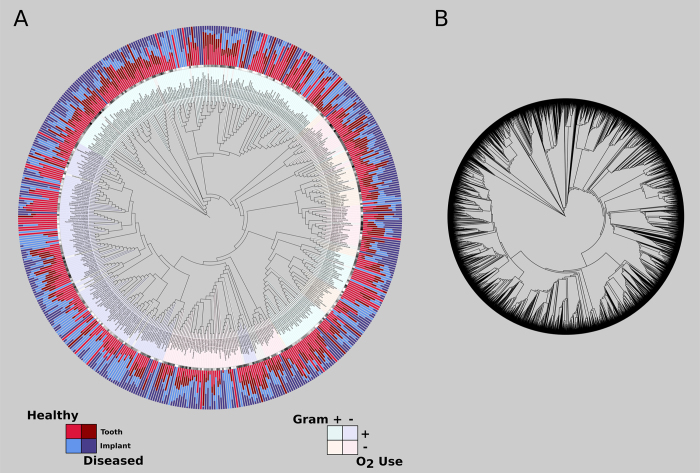
(**A**) Relative abundance of the species-level Operational Taxonomic Unit (sOTUs) data for the four groups of healthy and diseased periodontium and implants, created using PhyloToAST and iTol (legends added after export from iTol). The center of the figure is a circular phylogenetic tree representing the evolutionary relationships of the identified sOTUs. The sOTU labels (first ring outside the tree) are color-coded by Gram status and oxygen use. The outer ring represents the normalized mean relative abundance of the identified sOTUs from the peri-implant and periodontal biofilm samples^16^. This figure presents the clear differences between the microbiomes of teeth and dental implants, demonstrating that the two represent distinct ecological niches that cannot support identical species composition. (**B**) A phylogenetic tree rendered by iTol representing the same dataset from (**A**), but without having condensed the list of OTUs by species name using the OTU condensing workflow in PhyloToAST. The resulting tree in B has 6670 entries, while the tree in A has 206. Note that, due to the large size, iTol will not display the sOTU names as branch labels for the tree in (**B**) as is seen in (**A**).

81 dentate adult subjects with at least one tooth-bounded implant in function for at least one year were recruited for the study and categorized into the following groups: periodontal and peri-implant health, healthy periodontium adjoining diseased implant, diseased periodontium adjoining healthy implant, and both sites diseased. After quality filtering, 1.9 million 16S sequences were identified at 99% similarity against the Greengenes database. Comparing the tooth/implant pairs revealed that for all species, 60% of individuals shared less than 50% of species between the tooth and the adjoining implant. For highly abundant species (1.0% minimum relative abundance), 85% of individuals shared less than 8% of the microbiome between tooth and implant. Furthermore, the Shannon Diversity index revealed significantly higher diversity in the periodontal microbiome when compared to the peri-implant microbiome. Figure 6A is a visual representation of the relative abundances of species in the different environments in both health and disease. Together these data suggest that simple geographic proximity is insufficient as a determinant for microbial colonization; and that the periodontal and peri-implant ecosystems differ enough to produce distinct microbiomes. The trees seen in Fig. 6A,B are a new visualization available for QIIME-analyzed data through the PhyloToAST iTol.py script in conjunction with the Interactive Tree of Life (iTol) website^17^,^18^. The branch labels are genus/species names (where possible) extracted from the full taxonomy for each entry. The PhyloToAST script replaces the OTU codes in the QIIME-generated Newick-format tree file with those names making for a more useful representation. In Fig. 6A, the two data tracks immediately outside the ring of branch labels, represent the log base-10 overall abundance and the normalized mean relative abundance (between the four sample groups) for each entry on the phylogenetic tree. Figure 6B is a rendering of the same data without having run the OTU condensing workflow. The tree in 6B contains 6670 entries, whereas the tree in 6A contains 206. Note that iTol will not display the branch labels for the tree in 6B due to the large number of entries.

## Discussion

Our new software package, PhyloToAST, has been applied to three oral microbiome datasets: examining salivary and subgingival bacterial community compositions in smokers and never-smokers, an investigation of the rat model of periodontitis, and dental implant recipients. Compared to QIIME running on the basic Amazon EC2 instance, our pipeline running in parallel on the compute cluster at the Ohio Supercomputer Center reduced the processing time of identifying OTUs, for an average of 2 million 16S sequences, from over one week to less than nine hours; additionally compressing the process from two computationally intensive steps to one. Furthermore, we developed and applied algorithms to reduce single-primer bias (requires sequencing using two separate primers), condense redundant taxonomic output, and automatically generate the data for visualizing phylogenetic quantification of sample OTU abundance using the Interactive Tree of Life website^17^,^18^. Our tools integrate with the QIIME pipeline, are free and open source (MIT license), and available at http://phylotoast.org.

## Methods

### Human subject recruitment and sample collection

All protocols were approved by appropriate licensing committees and carried out in accordance with the approved guidelines.

#### Case Study 1

The study and all protocols were approved by the Office of Research and the Institutional Review Board at The Ohio State University and the NHS National Research Ethics Service Northeast. Two hundred periodontally healthy individuals between 21 and 40 years of age were recruited and written informed consent was obtained. Subjects who reported diabetes, HIV, pregnancy, immunosuppressants, bisphosphonates, steroids, current orthodontic therapy, antibiotics or professional dental cleaning within 3 months, and those requiring pretreatment antibiotic coverage were excluded. Subjects completed a demographic and tobacco exposure questionnaire and were examined by calibrated periodontists. Subjects had least 20 natural non-carious teeth, ≤3 mm probing pocket depths at all sites, plaque index of ≤0.9^19^ and gingival index of ≤1.2^20^. Both groups were frequency-matched for age, gender, race/ethnicity, education and socioeconomic status. Pooled plaque samples were collected by inserting endodontic paper points into the subgingival sulci of 15 teeth for 10 seconds. Samples were stored at −80 °C until further analysis.

#### Case Study 3

Approval for this study and all protocols was obtained from the Office of Research and the Institutional Review Board of The Ohio State University. Eighty dentate adults with at least 1 tooth-bounded dental implant in function for at least 4 years were recruited from those seeking care at the College of Dentistry and written informed consent was obtained. Exclusion criteria included diabetes, pregnancy, human immunodeficiency virus (HIV), use of immunosuppressant medications, bisphosphonates, or steroids, antibiotic therapy or oral prophylactic procedures within the past 3 months, need for antibiotic coverage before dental treatment, and fewer than 20 teeth present in the dentition. Tobacco exposure was assessed by questionnaire, and current smokers with a 10 pack-year history or greater were recruited. A diagnosis of implant health and disease was made according to the criteria delineated by the Consensus Report of the Sixth European Workshop of Periodontology^21^. Peri-implant samples and periodontal samples were collected by inserting endodontic paper points into adjoining peri-implant and periodontal crevices for 10 seconds. Samples were stored at −80 °C prior to preparation for sequencing.

### Animal sample collection: Case Study 2

Approval for this study and all protocols was obtained from the Institutional Animal Care and Use Committee of The Ohio State University, and all experiments were performed in accordance with relevant guidelines and regulations. Eight 1-year old female rice rats were anesthetized and teeth affected by severe periodontitis (Probe depths >3 mm, attachment loss >60% of root length, bleeding on probing) as well as healthy teeth (Probe depths <2 mm, attachment loss <10% of root length, bleeding on probing) were extracted and subgingival plaque collected using scalers. DNA was isolated, amplified, sequenced, and analyzed as described below.

### Sequencing and data analysis

A previously described methodology for DNA isolation was used^22^. Bacterial samples were removed from the paper points by adding 200 *μl* of phosphate buffered saline (PBS) and vortexing for 1 minute. The paper points were then removed, and DNA isolated using a Qiagen MiniAmp kit (Valencia, CA) according to the manufacturers instructions. Multiplexed bacterial tag-encoded FLX amplicon pyrosequencing was performed using the Titanium platform (Roche Applied Science, Indianapolis, IN, USA) as previously described^23^ in a commercial facility (MRDNALab, Shallowater, TX, USA). To summarize, 16S rRNA genes were amplified using a single step PCR with broad-range universal primers and 22 cycles of amplification; in addition to introducing adaptor sequences and sample-specific bar-code oligonucleotide tags into the DNA. Regions V1–V3 and V7–V9 of the 16S rRNA genes were sequenced using previously described primers^8^.

The data in Case Study 2 were analyzed with QIIME version 1.9 installed on the Ruby cluster at the Ohio Supercomputer Center (OSC). Case studies 1^9^ and 3^16^ are previously published datasets included to illustrate various aspects of PhyloToAST, and were orginally analyzed using QIIME version 1.6. Figure 2A,C were recreated from the original data using QIIME 1.9. For all datasets (including each separate primer set) a QIIME-compatible mapping file was created which lists for each sample, at a minimum, the following data: sample identifier, barcode sequence, linker/primer sequence, and description. Additional metadata were included where necessary. The raw 16S data was demultiplexed and filtered according to standard QIIME protocol.

Figure 1 summarizes the changes and additions introduced to the standard QIIME 16S processing pipeline; a description of the full process follows. With the quality filtered data, OTUs were identified by BLAST (Basic Local Alignment Search Tool)^24^ (v2.2.22 per QIIME requirements) using Greengenes v13.8^25^ or the Human Oral Microbiome Database (HOMD, v13.2)^26^. Sequences were compared to the database at a 99% similarity level. In order to perform this search efficiently, 16S datasets were split into chunks of approximately 20,000 sequences each (chosen to optimize processing time and available computational resources), using the split_sequence_data script (PhyloToAST). A Portable Batch System (PBS) job script was generated for each sequence data file (multi_parallel_pick_otus script, PhyloToAST) instructing that the QIIME parallel_blast_pick_otus script be run on the data, and all jobs were submitted to the PBS queuing system at the OSC with the multi_qsub script (PhyloToAST). Thus, each dataset was split across multiple machines to be run simultaneously, allowing the entire dataset to be processed in the same real-time as a single chunk. The resulting output files from each parallel run were merged into a single file containing all the results using the merge_otu_results script (PhyloToAST).

We have previously published results showing that use of a single 16S hypervariable region as a sequencing target introduces bias into the results by overrepresenting some genera while under representing others. By targeting two of the hypervariable regions, we capture more variety in the population, but must scale down the reported abundance for OTUs captured by both primers^8^. This process of primer averaging is implemented by PhyloToAST in the primer_average script which takes in the two separate primer-specific OTU result files from the previous step and returns a single averaged overall OTU result file.

The oral flora include many species that can be considered transient, or not part of the core community of interest during analysis. We therefore created a program to filter out, in an initial pass, OTUs that, when aggregated at the genus level (by default), occur in less than 5% of samples, and in a separate pass, remove aggregate OTUs that have an abundance of less than 0.01% of the overall sequence data. The removed OTUs, their percent abundance, and associated sequence IDs are output to a separate file for examination. We have obtained good results using these defaults on oral cavity samples to remove transient allochthonous organisms, but both parameters can be modified by the user if they are not appropriate for other sample sources. For the subsequent steps, it is not necessary to operate on the entire dataset of sequences; so a single representative can be chosen for each OTU. For processes such as generating phylogenetic trees, it is advantageous to have longer sequences, so instead of choosing a representative from the input data, we substitute the full-length 16S sequence from the Greengenes database. At this point, each sequence has been assigned to an OTU, but an additional file linking OTU IDs with the full taxonomic classifier is needed. QIIME only provides further computationally intensive methods for doing so (such as the Ribosomal Database Project classifier), however, since we perform OTU picking by BLAST, these associations can be done by a simple database lookup, and PhyloToAST provides this functionality in the assign_taxonomy_by_blast_result script.

### OTU Condensing Workflow

Databases such as Greengenes often contain many species variants of 16S genes, which, for some analysis modalities (visualization for example) create unnecessary clutter. In PhyloToAST we have implemented a workflow program that condenses the OTU results in three steps by joining on full taxonomic identifiers and binning sequence data by those identifiers. The final step of processing the 16S sequence data is to collect, in tabular format, the total sequence abundance by OTU and originating sample. The QIIME script make_otu_table performs this operation, outputting to the generic BIOM (BIological Observation Matrix) format (http://biom-format.org), which PhyloToAST also makes extensive use of. Finally, many analysis modalities require that OTUs be grouped according to evolutionary relationships into a phylogenetic tree. We used the three step method provided by QIIME to align sequences, filter the alignment via lanemask, and finally construct a phylogenetic tree.

### Visualization

From these starting points (BIOM table and phylogenetic tree) we produce the rest of our visualizations and analyses. One of the most common analyses in ecology (microbial and otherwise) is alpha diversity. Calculation of this metric provides insight into the abundance and range of species in a specific environment and has been approached in a wide variety of methods (the biodiversity software package, EstimateS, provides 51 different metrics). PhyloToAST provides a tool that calculates several alpha diversity metrics (using the array of metrics provided by scikit-bio, available at http://scikit-bio.org/), visualizes the results as a set of per-group histograms smoothed by kernel density estimation (see Fig. 4), and provides basic statistical comparisons between the plotted groups.

Another common analysis method is beta diversity. This metric is a means of quantifying the difference or change in species between multiple ecosystems, and is generally calculated alongside alpha diversity. QIIME provides a script (beta_diversity_through_plots) to calculate beta diversity difference metrics (phylogenetic differences if a tree is provided) and visualize the results. The high-dimensional result data are typically visualized using some multidimensional or non-metric scaling tool. QIIME includes two tools that visualize this data using Principle Coordinates Analysis (PCoA): A set of automatically generated 2D plots enumerating all combinations of the first three principle coordinates for all category groupings listed in the mapping file, and a 3D interactive web-based tool (Emperor)^27^. PhyloToAST provide a more aesthetically pleasing 2D and 3D (interactive) PCoA plotting tool with many customization options. In addition, a separate bubble-plot version of the PCoA is available that sets the plot point sizes by the relative abundance of a specified OTU (see Fig. 3). Both tools produce publication-ready images.

An alternative dimensionality-reduction technique commonly used in other disciplines, but previously applied to microbial composition data^28^, is Linear Discriminant Analysis (LDA). This technique is similar to Principle Components Analysis (PCA) and PCoA in that it finds linear combinations of input variable that best explain the data, but LDA explicitly makes use of class labels in its modeling to explain differences. PhyloToAST provides a tool similar to the PCoA tools for calculating and visualizing the results of the Linear Discriminant Analysis (see Fig. 5).

The final visualization tool we provide integrates with The Interactive Tree of Life (iTol) website (itol.embl.de)^17^,^18^, which provides an extremely useful circular visualization of phylogenetic trees and data connected to them. Unfortunately it requires all of the data to be pre-calculated and set in a specific format. We have provided with PhyloToAST a program that calculates several types of abundance metrics from a BIOM table: log-transformed raw abundance, Mean Relative (per sample) Abundance (MRA), and Normalized (by metadata category) MRA; with optional variance stabilization by arcsine square-root transformation. Each calculation mode produces an iTol-compatible dataset for visualization around a phylogenetic tree (see Figs 6 and S1).

### Code and Documentation

All code for PhyloToAST is made available free and open source under the MIT license. The code is hosted on GitHub and may be downloaded at http://github.com/smdabdoub/phylotoast. Documentation of the API and all executable scripts can be found at http://docs.phylotoast.org.

## Additional Information

**How to cite this article**: Dabdoub, S. M. *et al*. PhyloToAST: Bioinformatics tools for species-level analysis and visualization of complex microbial datasets. *Sci. Rep.* **6**, 29123; doi: 10.1038/srep29123 (2016).
